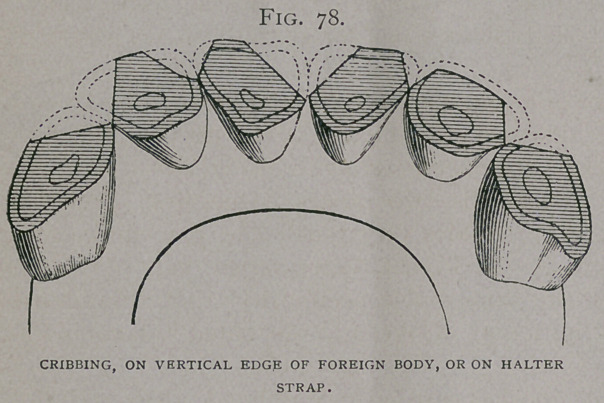# Age of the Horse, Ox, Dog, and Other Domesticated Animals

**Published:** 1891-07

**Authors:** R. S. Huidekoper

**Affiliations:** Vet.


### AGE OF THE HORSE, OX, DOG, AND OTHER DOMESTICATED ANIMALS.

BY R. S. HUIDEKOPER, M.D., VET.

[*Continued from page 285.*]

### ACCIDENTAL IRREGULARITIES.

Accidents from various causes, injuries, wounds, fractures of the jaw bone, with or without loss of the teeth, and abnormal growths, may so interfere with mastication on one side of the mouth, that they cause an excessive wearing of the teeth on the other side.

Vicious, nervous and irritable horses frequently bite at their mangers, chains, wagon pole or other foreign bodies and chip off the edges of the incisors, or they may even wear them to a considerable degree, so as to greatly change their form and tables, and interfere more or less with the characters from which we judge the age. In any of these cases, however, the cause is evident, and the unilateral wearing, or the rough, irregular loss of substance can be replaced by the imagination, and the accurate age determined.

IRREGULARITIES FROM CRIBBING.

Cribbing is recognized as of two kinds: 1st, that of the wind sucker who pursues the habit, nose in air, and consequently produces no abnormal wearing of the teeth; and 2d, the cribber, who requires some foreign body between its teeth, and wears them at the point of prehension. For the former M. Goubaux proposed, in 1866, the name of "æropinic."

According to the manner of cribbing, and the character of the object, which the horse chooses for support of the teeth, the wearing of the latter may be much varied. The object seized may be the feed box, the rail of the manger, or a part of the stall, which may be horizontal or may be vertical; it may be the end of a poll or shaft, a chain, hitching strap or part of another horses harness; in one case of the writers, the horse would only crib on a small piece of wood when hung loose on a cord. Rare cases crib by seizing their own legs. Sometimes the support is only taken with the lips or the tuft of the chin, and in these there is no wearing of the teeth.

According to the size of the object, or the position assumed by the horse in cribbing, the contact of the teeth may be only by the anterior borders, only by the posterior borders, or by both; it may be by one jaw or by both; it may be by a number of teeth, or only by one or two teeth. In whatever manner the cribbing is done, but little force is used, and the worn surface of the teeth is smooth, even and polished, so that it is readily distinguished from the roughened edges of teeth worn by vicious or nervous biting. When the support of the teeth has been taken, the animal makes an effort of deglutition, which is followed by a peculiar "cluck" sound. In examining an animal, even where the age is readily recognized, care should be taken to open the jaws completely so as to inspect all surfaces of the teeth. While cribbing marks, when on the anterior face of the teeth, are apparent on superficial examination, those on the posterior face, are often hidden by the foam and saliva, unless care is taken to wipe the latter away. A slight bevel worn on the

F.G. 75.

CRIB MARKS, BY PRESSURE OF THE TEETH AGAINST FOREIGN BODIES.

FIG. 76.

CRIB MARKS, BY SEIZING FOREIGN BODIES INSIDE OF THE JAWS.

FIG. 77.

CRIB MARKS.—A, THE REVERSE OF FIG. 76, D. B, C, AND D, WEARING OF WHOLE TABLE, UPPER, LOWER OR BOTH.

anterior surface is evident, with the teeth closed, from the separation of the enamel, and the presence of a yellow line, made by the exposed dentine, while a considerable bevel might be overlooked when its surface is a continuation of the yellow dentine of the table and is looked at from in front.

When the animal cribs by pressing the incisors against some foreign body, the wearing takes place on the anterior face of the teeth (Fig. 75, A, B, C, D).

When the object is seized in the mouth it may wear only the posterior faces, (Fig. 76, A. B. C.); or if a turn board or similar body is held, it may wear the anterior borders of one jaw, and the posterior of the other, Fig. 76, D and Fig. 77 A.)

If a thin object is seized evenly between the jaws, the tables of the upper or the lower, or of both sets of incisors, may be worn. In the latter case the age must be determined by the thickness of the maxilla, the obliquity of the shortened teeth and their size as they emerge from the gums. (Fig. 77, B. C. D).

FIG. 78.

CRIBBING, ON VERTICAL EDGE OF FOREIGN BODY, OR ON HALTER STRAP.

Fig. 78, shows the wearing produced by vertical objects, halter straps, which the animal renders tense by backing to its full length, wires, small chains, etc.

Most horses crib only on a given character of object, and when removed to another stall, or fastened so that they cannot reach the regular resting place, may stop the habit for a time or be broken of it entirely. Others will sooner or later find some other body that suits them and recommence, so that the same animal may have two sorts of crib markings. Others, when tied up, will learn to crib in the air.

The rasp and file are sometimes used by dealers to shorten the incisors, and, while adding a little to the apparent age of the animal, remove the bevel which shows the habit of cribbing. A careful examination will always show a roughened surface, however, unlike the smooth polish of a naturally worn table.

[TO BE CONTINUED.]

## Figures and Tables

**Fig. 75. f1:**
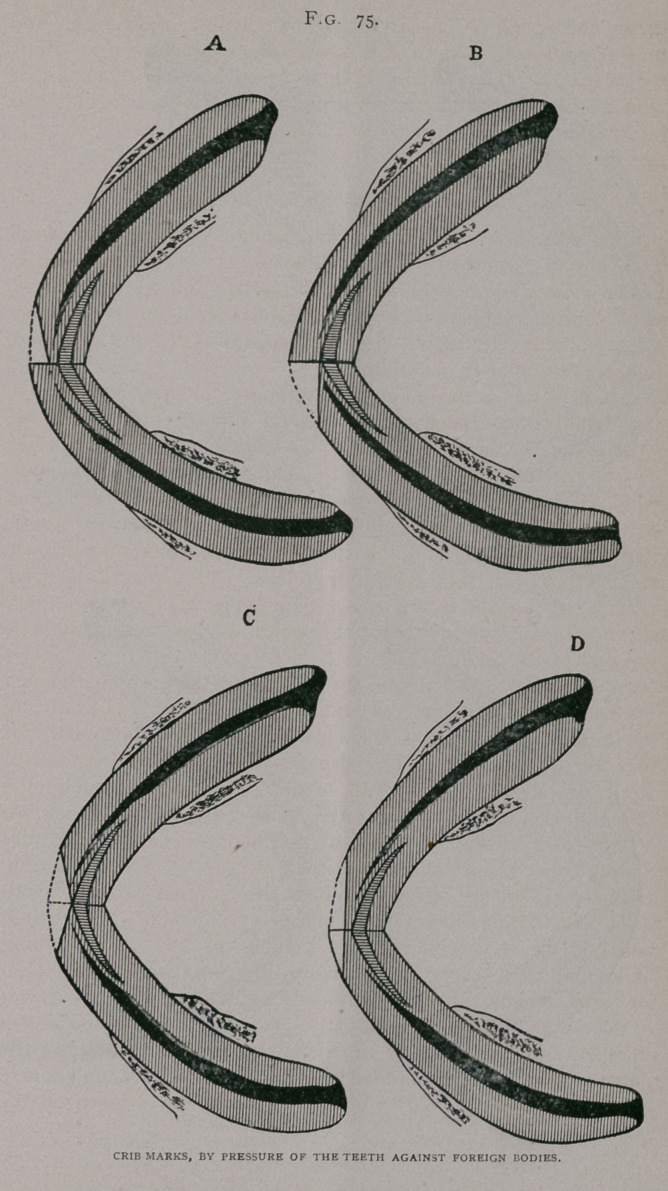


**Fig. 76. f2:**
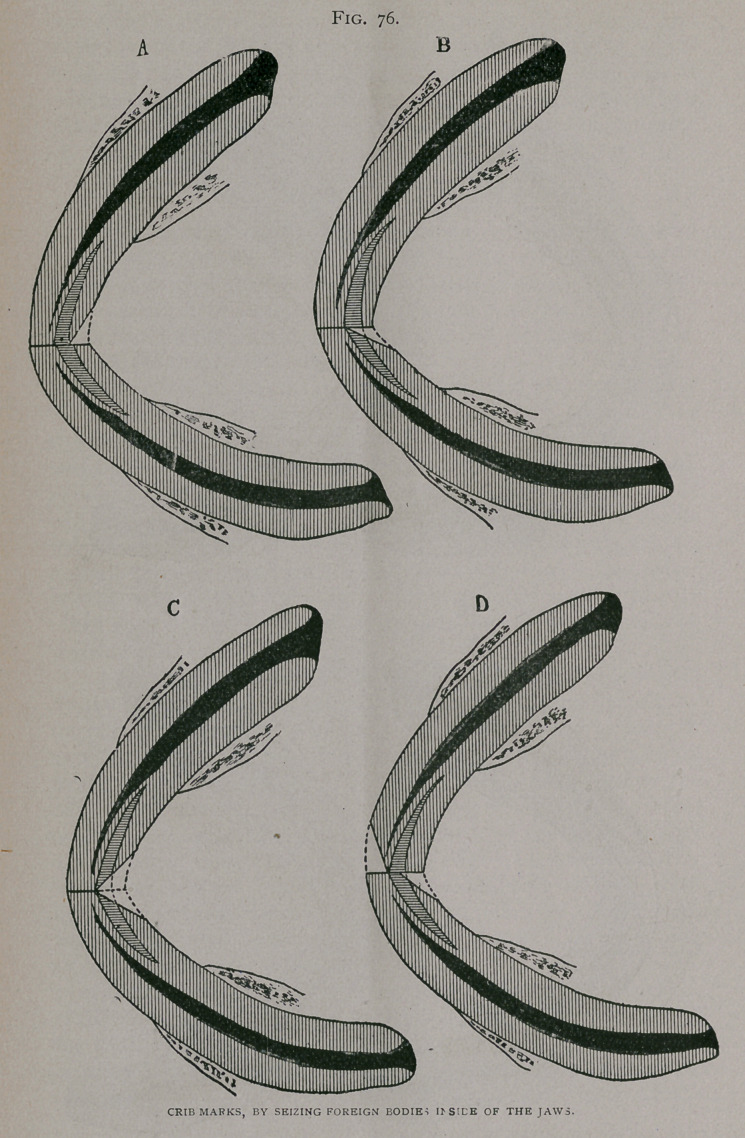


**Fig. 77. f3:**
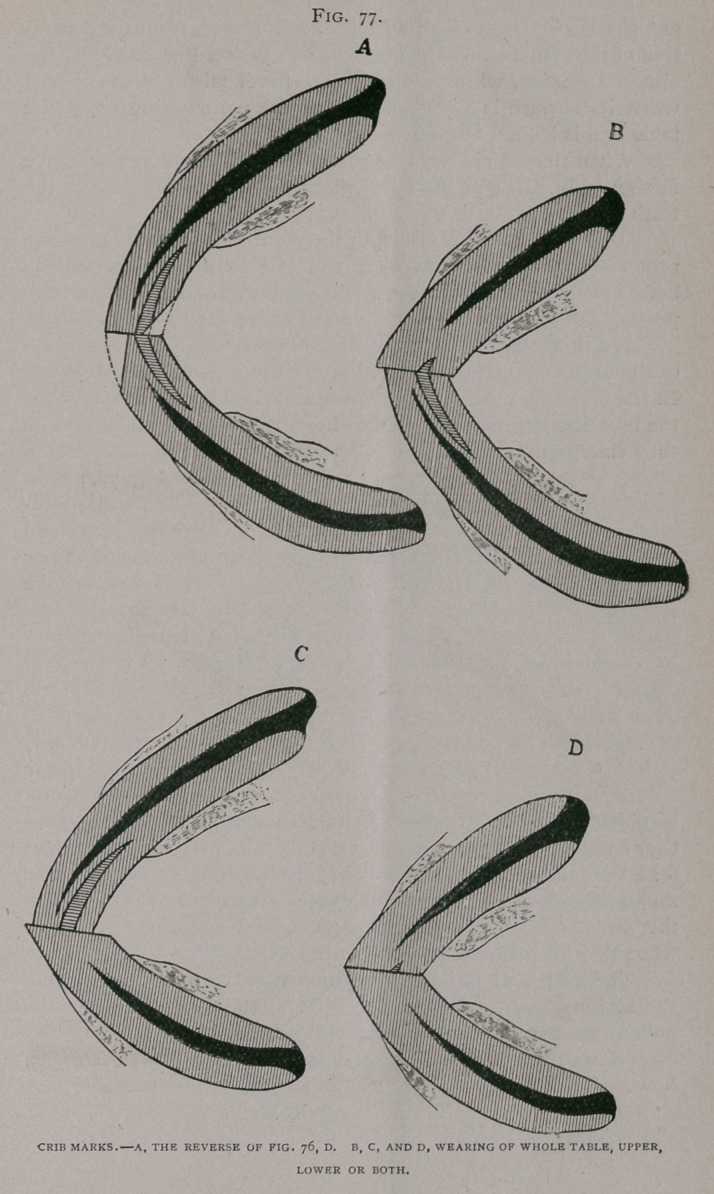


**Fig. 78. f4:**